# Evidence for lung epithelial stem cell niches

**DOI:** 10.1186/s12861-015-0082-9

**Published:** 2015-09-16

**Authors:** Matt L. Donne, Andrew J. Lechner, Jason R. Rock

**Affiliations:** Department of Anatomy, University of California, San Francisco, USA

## Abstract

Recent studies have identified epithelial stem and progenitor cell populations of the lung. We are just beginning to understand the mechanisms that regulate their homeostatic, regenerative and maladaptive behaviors. Here, we discuss evidence of regulatory niches for epithelial stem cells of the lung.

## Background

Unfortunately, curative therapies do not exist for many end stage lung diseases and the final option is often a full lung transplant. The success of this procedure is limited by an inadequate supply of suitable donor organs and chronic allograft rejection in recipients. In vitro studies and animal models have demonstrated that the lung, like some other solid organs, has an endogenous capacity for maintenance and repair (reviewed in [1]). One potential alternative strategy for the management of lung disease would be to harness this reparative potential to prevent or reverse the debilitating effects of pathologic remodeling of the lung. This will require a better understanding of the stem/progenitor cell populations and the cellular and molecular mechanisms that regulate their behaviors.

## Lung structure and cellular composition

The cellular composition of the epithelial lining of the respiratory tract varies along its proximo-distal axis [2]. The conducting airways from the trachea to bronchioles of human lungs consist of pseudostratified epithelium, comprising roughly equal proportions of basal cells, secretory cells, and ciliated cells, as well as some neuroendocrine cells. The smallest bronchioles, known as terminal and respiratory bronchioles, are lined with a simple columnar or cuboidal epithelium containing secretory and ciliated cells with fewer basal cells. The epithelia of these conducting airways form a tight barrier against the outside world and are specialized for the process of mucociliary clearance. The alveoli are lined by type 1 and 2 alveolar epithelial cells, called AEC1 and AEC2, respectively, hereafter. These cells are also specialized for barrier function and the extremely thin AEC1s share a basement membrane with the surrounding network of pulmonary capillaries to facilitate the diffusion of gases between the atmosphere and the circulation.

This general distribution of epithelial cell types is conserved between humans and model organisms such as rodents. However, there are notable differences [2]. For example, the transition from a pseudostratified to columnar epithelium occurs more proximally in rodents, so only the trachea and mainstem bronchi are lined with a pseudostratified epithelium. Nearly all intralobar airways in mice are lined with a simple columnar or cuboidal epithelium with few, if any, basal cells. In mice, the abrupt transition from a conducting airway to the alveoli it supplies is known as a bronchioalveolar duct junction. In humans, terminal bronchioles give rise to respiratory bronchioles from which many alveolar ducts terminate ultimately in alveoli [3].

## Stem cell populations in the lung

Unlike some other organs, the lung is relatively quiescent under steady state conditions [4]. Relatively infrequent progenitor cell divisions maintain the respiratory epithelium. For this reason it is common to experimentally induce cell turnover in order to study stem cell behaviors and clonal dynamics in the lung [5, 6]. As a result, relatively little is known about cell lineage relationships and stem cell niches under state conditions. Moreover, because assays for human lung stem cells are only just gaining popularity, our understanding of lung maintenance and repair is primarily based on studies from model organisms. Here, we discuss recent advances toward the identification of stem cell populations in the lung and their putative niche components, highlighting species differences and experimental design where appropriate.

It is generally accepted that under steady state conditions and in response to mild injury, distinct epithelial stem cell populations maintain and repair each of the lung regions described above (reviewed in [1, 7]). Basal cells of the pseudostratified conducting airway epithelium characteristically express the transcription factor Trp63, cytokeratin 5, podoplanin, NGFR and, variably, cytokeratin 14 [2, 8]. Early studies showed that basal cells purified from donor rats are capable of generating a pseudostratified epithelium comprised of basal, ciliated and secretory cells when seeded into a denuded trachea grafted subcutaneously in a host [9]. This suggested that basal cells, as a population, are capable of self-renewal and differentiation. More recently, in vivo genetic lineage tracing studies in mice and humans have shown that basal cells are capable of long-term self-renewal and the generation of secretory and ciliated cells [8, 10, 11]. Finally, individual p63+ basal cells from either mouse or human lungs can be cultured in Matrigel to generate multicellular tracheospheres (or bronchospheres) that are made up of basal cells and Krt8+ luminal cells (including secretory and ciliated cells) [8, 12]. Emerging data suggest that basal cells are heterogeneous, at both the transcriptional and functional levels [6, 13]. The degree to which this heterogeneity is intrinsic or a function of microenvironmental cues is not known.

Secretory Club cells (previously known as Clara cells, [14]), characterized by apical protrusions packed with secreted proteins including SCGB1A1, are also found in the conducting airways. In simple columnar and cuboid epithelia where basal cells are rare or absent, evidence from lineage tracing in mice suggests that Club cells are capable of long-term self-renewal and the generation of ciliated and mucous-producing goblet cells under steady state conditions and in response to injury [15, 16]. Club cells of human airways proliferate under steady state conditions [17], but the extent of their capacity for self-renewal and differentiation is not known.

In the alveoli, AEC2s are the primary source of surfactant-associated protein C (Sftpc), a component of the layer of surfactant that reduces surface tension to prevent alveolar collapse. Over 40 years ago, it was reported that AEC2 proliferate in response to injury in rodents, suggestive of a role in repair [18, 19]. More recently, genetic lineage tracing experiments have provided support for a model in which AEC2s are capable of long-term self-renewal and the generation of AEC1 in both alveolar regeneration and under steady state conditions [20–22]. AEC2s purified from human lungs and cultured on plastic or in 3D with fibroblasts have the ability to proliferate and give rise to cells with some characteristics of AEC1 [20, 23]. These data suggest that AEC2s also function as a stem cell population in human lungs.

Recent data suggest that there are exceptions to the compartmentalization of lung epithelial cell lineage relationships described above. In the trachea, cells expressing Scgb1a1 are capable of “de-differentiating” into basal cells following depletion of basal cells using SO_2_ or genetic ablation with diphtheria toxin [16, 24]. Lineage tracing studies have also shown that Scgb1a1+ cells, perhaps airway Club cells, can give rise to alveolar lineages following severe injury with bleomycin [20, 22, 25, 26]. The bronchio-alveolar junction harbors a putative progenitor called the bronchioalveolar stem cell or BASC that can give rise to both airway and alveolar lineages in vitro and under some injury conditions [27]. To date, no human counterpart of this cell type has been identified and its contribution to maintenance and repair in vivo awaits genetic lineage tracing. Following very severe lung injury in mice caused by infection with a murinized version of the pandemic H1N1 strain of influenza, “pods” of cells expressing the airway markers p63 and Krt5 are observed in the alveolar region [28]. The origin and differentiation potential of these pods in vivo is not clear [28–31]. Finally, in the alveoli at least a subset of AEC1, long thought to be post-mitotic, appear to be able to generate AEC2 under regenerative conditions [32]. Because these data were obtained in mouse models of injury/repair, their relevance to human lung maintenance and repair are not clear.

## Putative niche components

Considerable progress has been made toward identifying the signals that regulate lung epithelial stem cell self-renewal and differentiation. These include Notch, Hippo/Yap, ROS/Nrf2, EGF, FGF, c-myb, and cytokines including IL-4, -13 and -6 [13, 29, 33–40]. Neighboring epithelial cells, stromal cells (including fibroblasts, smooth muscle cells, and endothelium) and immune cells all represent potential sources for these factors. Here, we discuss some data to support each of these as components of the lung epithelial stem cell niche.

### Niche of the pseudostratified airway epithelium with basal cells

**Fig. 1:**
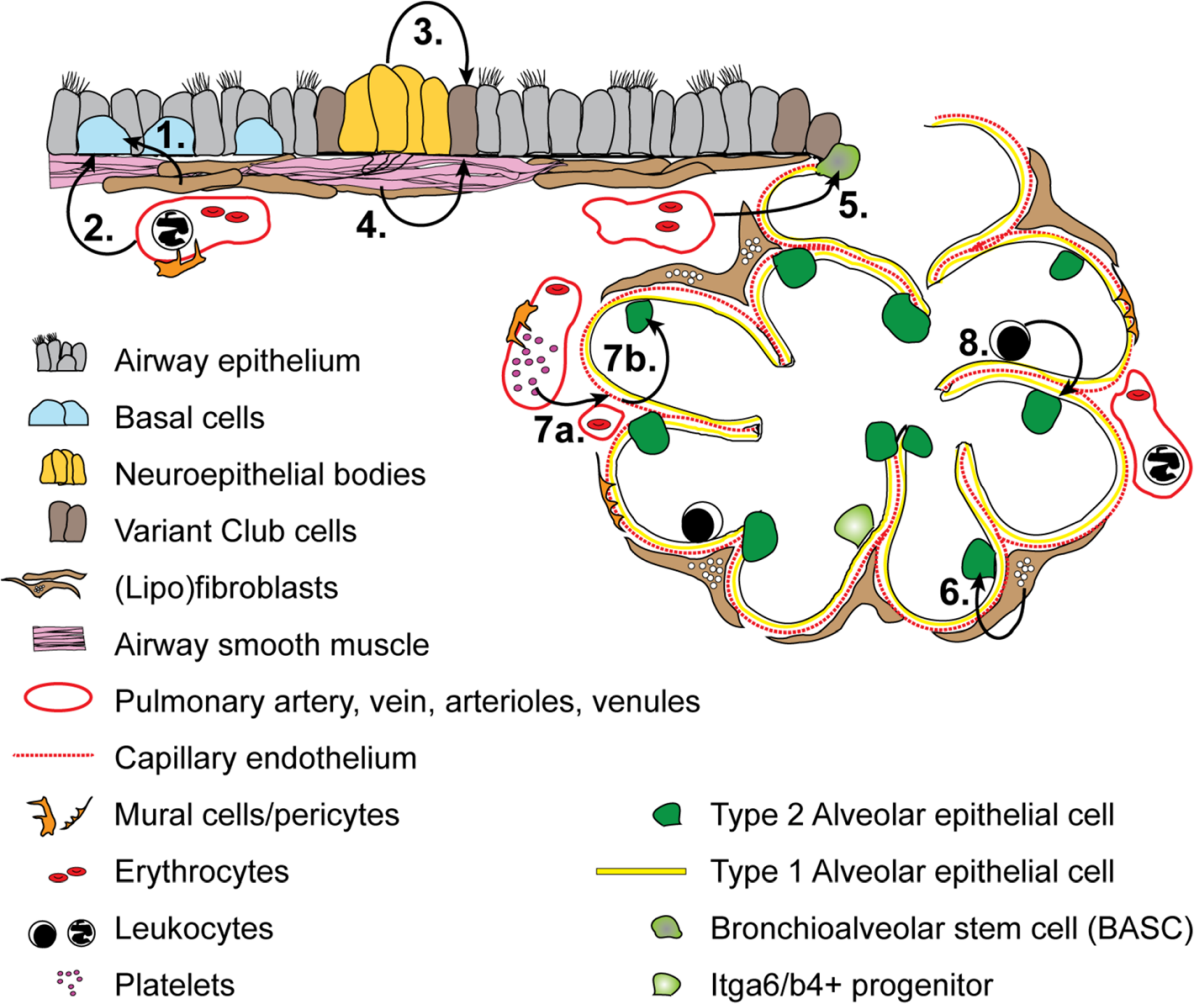
Selected putative niche-stem cell interactions in lung repair/regeneration. **1** Fibroblasts upregulate the expression of IL-6 following injury of the airway epithelium to promote ciliated cell differentiation from basal stem cells in mice [38]. **2** Human airway basal stem cells secrete VEGF to simulate endothelial cells that, in turn, support basal stem cells [43]. **3** Variant Club cells are found adjacent to neuroepithelial bodies and repopulate simple columnar airway epithelia following injury in mice [46–48]. **4** Parabronchial smooth muscle cells secrete FGF10 following airway injury to promote the activation of variant Club cells in mice [51]. **5** BASCs secrete Bmp4 to activate calcineurin/NFAT-c1 signaling in endothelial cells that, in turn, secrete Tsp1 to promote alveolar differentiation of BASCs in mice [53]. **6** (Lipo)fibroblasts are required for the growth of mouse and human AEC2 in vitro [20]. Evidence suggests that these cells give rise to myofibroblasts that are critical for compensatory lung growth following pneumonectomy [57]. **7a**. Platelet derived SDF-1 promotes the production of MMP14 in endothelial cells following pneumonectomy in mice [64]. **7b** MMP14 activates EGF-like growth factors sequestered in the matrix to promote the activation of AEC2s [63]. **8** Myeloid cells are critical for the resolution of bleomycin-induced pulmonary fibrosis in mice [70]. The proliferation of AEC2 and compensatory growth following pneumonectomy is impaired in mice with insufficient numbers of myeloid cells in the lung ([72] and our unpublished data)

Basal cells of the surface epithelium that lines the airways are capable of long-term self-renewal and differentiation [8]. However, a population of label retaining basal cells is localized to the submucosal glands of the large airways [41, 42]. These cells are protected from inhaled noxious gases, particulates and microorganisms, therefore representing a reserve to repopulate the airways following extreme injury. Their location near the basal lamina preferentially exposes basal cells to factors secreted by the underlying stromal cells. A recent report showed that basal cell differentiation into ciliated cells is enhanced by IL-6/Stat3 signaling [38]. Following injury of luminal cells by inhalation of SO_2_, surviving basal cells self-renew and differentiate into ciliated and secretory cells to repopulate the epithelium. During this regenerative response, subepithelial PDGFRA+ fibroblasts upregulate the expression of IL-6, presumably directing the differentiation of basal cells [38] and Fig 1. Another study investigated subepithelial endothelial cells as a potential niche component of isolated human airway basal cells [43]. Transcriptional profiling data and in vitro assays support a model in which airway basal cells express VEGF that activates MAPK in endothelial cells via VEGFR2. The endothelial cells, in turn, support the growth of basal cells [43] and Fig 1. Whether changes in this axis are altered in disease or during repair is not known, but could represent a new therapeutic approach for the management of airway disease. Importantly, basal cells are capable of forming tracheospheres in Matrigel with growth factors independent of supporting cells types, although addition of lung fibroblasts and endothelium can enhance colony forming efficiency [8, 12, 38, 40].

Several lines of evidence suggest that neighboring epithelial cells are components of the lung epithelial stem cell niche. For example, secretory cells (but not basal cells) divide to repopulate the airway epithelium following the depletion of ciliated cells; however, basal cells repopulate the epithelium following depletion of secretory and ciliated cells of the trachea [8, 16, 44]. Together, these data suggest that surviving epithelial cells communicate the degree and kind of injury to stem cells so that an appropriate regenerative response is mounted. The molecular mediators of this feedback loop are not known. A recent report provided evidence that this signaling is bidirectional. Basal cells supply a Notch signal to their daughter secretory cells. In the absence of this maintenance signal, secretory cells terminally differentiate to generate ciliated cells [45].

### Niche of the mouse intralobar airways and alveoli

#### Airways

Clusters of neuroepithelial cells, known as neuroepithelial bodies (NEBs), represent another putative niche within the airway epithelium Fig 1. A population of injury resistant “variant” Club cells localizes to these clusters and repopulates the airway epithelium following depletion of Club cells by administration of the polycyclic aromatic hydrocarbon naphthalene [46–48]. There is evidence that calcium signaling in Club cells is induced by the secretion of ATP from NEBs [49]. How this and other NEB-derived signals affect club cell survival, quiescence, proliferation, and differentiation is not known. During embryonic development, the specification of Club cell precursors is dependent on Notch signaling from the NEBs [50]. Several studies have reported a role for FGF10 secreted by parabronchial smooth muscle cells or resident mesenchymal stromal cells in the activation of epithelial stem cells, including Club cells [40, 51, 52] and Fig 1.

#### Most distal terminal airways

The diversity of cell types and complex 3D organization of the terminal airways and alveoli complicate the characterization of the epithelial stem cell niche in these regions in vivo. One way of overcoming this problem is to exploit in vitro assays. Genetic and pharmacological manipulation of candidate pathways as co-culture experiments allow for the efficient identification of niche components, signaling cascades, and cell:cell interactions that would be tedious in mice. However, the interpretation of this type of experiment must be cautious, as isolation can change cellular phenotypes and culture conditions, including growth factors and substrate, profoundly affect cellular behaviors.

The growth and differentiation of BASCs in vitro requires the presence of fibroblast feeder cells or lung endothelial cells, implicating these cells as niche components [27, 53] and Fig 1. Co-culturing BASCs with Tsp1 null endothelial cells significantly reduced the differentiation of BASCs toward alveolar lineages. This study went on to provide data to support a model in which Bmp4, expressed in epithelial cells, induces calcineurin/NFATc1 signaling and Tsp1 expression in nearby endothelial cells. Endothelial Tsp1, in turn, stimulates the alveolar differentiation of BASCs [53]. Mice homozygous for a null allele of Tsp1 showed impaired regeneration following treatment with bleomycin, suggesting that this pathway is active in vivo.

#### The alveoli

Most models used to study cell lineage relationships in the distal lung induce significant acute lung injury and disrupt the intricate cell-cell interactions of the alveoli. One very promising approach to interrogate the alveolar niche is to study compensatory regeneration following partial pneumonectomy (PNX), the removal of one of more lung lobes. This is a well-established model of adult alveologenesis that stimulates activation and proliferation of alveolar niche components without inducing acute lung injury [54, 55]. In this model, AEC2s are the major proliferating epithelial cell type [56]. Recent studies in the mouse identified a subset of PDGFRA+ fibroblasts in the alveolar niche that increase in number and differentiate into myofibroblasts post-PNX [57]. Blocking PDGFRA+ fibroblast function or differentiation through FGFR2 inhibition or targeting the PPARγ pathway impaired alveolar septation and compensatory growth post-PNX. Therefore, similar to their function in embryonic lung development, PDGFRA+ fibroblasts are essential niche cells during PNX-mediated lung regeneration [58, 59] and Fig 1.

In vitro co-culture of mouse or human AEC2s with lung fibroblasts generated clonal and proliferating “alveolar-spheres,” consisting of an internal layer of cells with AEC1 morphology and marker expression, and an external layer of surfactant-producing AEC2-like cells indicative of self-renewal and differentiation [20]. Importantly, AEC2s were unable to form spheres or survive unless fibroblasts were intimately associated with them, further implicating fibroblasts as a critical support cell for AEC2 [20, 40]. Pharmacologic manipulation of the FGF pathway in these cocultures suggests that this pathway promotes the differentiation of alveolar lineages from lung epithelial stem cells at the expense of airway lineages [40]. Beyond potential trophic factors derived from fibroblasts, these cells are also a source of extracellular matrix that modulates alveolar stretch and recoil. Data from a number of systems, including the lung, have shown that biomechanical forces are potent modulators of stem cell behaviors. This will be important to consider for the improvement of biomimetic and bioengineered lung replacements [60, 61]. The heterogeneity of fibroblasts and the cell lineage relationships amongst stromal cells of the lung are exceptionally poorly understood.

There is evidence that alveolar epithelial cells communicate with the surrounding endothelium. During branching morphogenesis, distal lung endoderm stimulates development of the capillary plexus, which, in turn, stimulates endodermal proliferation and alveolar septation [62]. During lung regeneration following partial PNX, inhibition of either VEGFR2+ or FGFR1+ signaling in pulmonary capillary endothelial cells (PCECs) impaired proliferation of both BASCs and AEC2s [63] and Fig 1. This group found that PCECs secrete matrix metalloproteinase 14, which activates EGF-like growth factors sequestered in the surrounding matrix. This subsequently stimulates AEC2 proliferation by activating EGFR. Importantly, restoring EGF signaling in animals with defective vasculature rescued defects in lung regeneration following PNX. This suggests that the regenerative effect of vasculature is more than simple perfusion. Recently, they showed that upstream stromal-cell-derived factor-1 (SDF-1, also known as CXCL12) secreted from platelets primes endothelial cell production of MMP14 during PNX-induced alveologenesis [64].

Numerous immune cell types populate the alveolus and lie in close proximity to AEC2s. Myeloid cells of the lung, including alveolar macrophages and neutrophils, promote alveologenesis during development and protect the epithelium throughout life by clearing proteinaceous debris from airway lumens, battling pathogens that escape the mucociliary escalator, and modulating the inflammatory milieu [65–69].

In the mouse model of bleomycin-induced lung fibrosis, macrophage depletion with clodronate during fibrotic stages of the model ameliorated the phenotype, while depletion of these cells during the recovery stage of the injury made it worse [70]. This effect was attributed to loss of alternatively activated M2 macrophages during recovery, but the molecular mechanisms by which these cells promote repair in the lung are not currently known. In compensatory growth following PNX, alveolar macrophages proliferate and upregulate pro-angiogenic and matrix remodeling genes [71]. Researchers also identified an increase in CD11b + myeloid cells in regenerating lung tissue post-PNX and provided some evidence that CD18-deficient mice, which suffer impaired leukocyte trafficking, have impaired generation of new lung tissue post-PNX [72] and Fig 1. Our own unpublished data show that macrophage number peaks together with epithelial proliferation and that depletion of macrophages impairs the proliferation of AEC2 post-PNX. These studies are beginning to probe how immune cells affect alveolar epithelial stem cells, but the identities of specific immune cell populations and their regenerative niche signals remain largely unknown.

## Conclusion

As our understanding of progenitor populations within the lung epithelium improves, we can begin elucidating the signals that regulate epithelial stem cell behavior. Animal model systems have enabled controlled studies of both progressive and acute lung injury where relevant regenerative and maladaptive mechanisms can be probed and manipulated. Furthermore, in vitro model systems have allowed reconstitution of human epithelial progenitor niches to facilitate testing of mechanistic hypotheses. We have highlighted some important niches for several lung epithelial progenitors. Further elucidation of lung stem cell niches and the signals with which they regulate progenitor cell behaviors have the potential to lead to improved targeted therapies to prevent or reverse pathological remodeling in lung injury and disease.
